# Calcium-dependent protein kinases in plant immunity: from calcium signaling to network integration

**DOI:** 10.3389/fpls.2025.1704615

**Published:** 2026-01-15

**Authors:** Lu Deng, Simin Ji, Gaopeng Wang, Xiang Liu

**Affiliations:** 1 School of Ecological Technology and Engineering, Shanghai Institute of Technology, Shanghai, China; 2 State Key Laboratory for Quality Assurance and Sustainable Use of Dao-di Herbs, Beijing, China

**Keywords:** calcium-dependent protein kinase, protein function, disease resistance, plant immunity, signaling pathway

## Abstract

In response to environmental stimuli, plants rapidly activate calcium signaling to initiate downstream responses. The transmission of calcium signals involves three primary processes: perception, decoding, and relay. Calcium-dependent protein kinases (CDPKs/CPKs), as key Ca^2+^ sensors, not only detect calcium signals but also respond to them by translating these signals into physiological activities within the cell. The influx of calcium ions (Ca^2+^) induced by pathogens triggers the conformational activation of cytosolic CPKs, which in turn enables the regulation of reactive oxygen species (ROS) production, MAPK cascades, transcriptional reprogramming, and hormone signaling. Acting as pivotal hubs in signal transduction, CPKs integrate diverse pathways to fine-tune the balance between growth and defense. We synthesize recent advances in understanding CPK-mediated immune mechanisms and their molecular crosstalk with other signaling networks. By highlighting emerging discoveries and unresolved questions, we provide a conceptual framework for exploiting CPKs to enhance durable and broad-spectrum disease resistance in crops.

## 1 Introduction

Plants are constantly challenged by diverse environmental stresses, yet their sessile lifestyle necessitates the evolution of intricate defense systems. Over long-term coevolution with pathogens such as oomycetes, fungi, viruses, bacteria, and nematodes, plants have developed multi-layered immune mechanisms capable of restricting pathogen invasion and mitigating damage (Kemen and Jones, 2012). These defenses can be broadly categorized into constitutive resistance, which relies on preformed structural barriers and antimicrobial compounds, and induced resistance, which is activated upon exposure to pathogens or elicitors (Kempel et al., 2011). At the core of induced immunity are two interconnected layers: Pattern-Triggered Immunity (PTI), initiated by Pattern Recognition Receptors (PRRs) sensing conserved pathogen-associated molecular patterns, and Effector-Triggered Immunity (ETI), which recognizes specific pathogen effectors through nucleotide-binding and leucine-rich repeat receptors (NLR) proteins, often eliciting a stronger and more durable response (Chisholm et al., 2006; Bigeard et al., 2015). Together, these strategies establish a dynamic immune system that enables plants to balance effective defense with continued growth. Importantly, both PTI and ETI rely on rapid intracellular signaling events, among which calcium signaling is particularly critical for ensuring specificity and amplification of immune responses.

Calcium ions (Ca^2+^) function as ubiquitous second messengers that orchestrate diverse aspects of plant physiology and immunity. Upon stress perception, transient fluctuations in cytosolic Ca^2+^ levels—referred to as “calcium signatures”—are generated, characterized by distinct amplitude, duration, and subcellular localization (Dodds and Rathjen, 2010). These signatures are decoded and propagated through a stepwise process involving initiation, interpretation, and relay of Ca^2+^ signals, which subsequently activate downstream defense pathways (Mahajan et al., 2008; Zeng et al., 2023). Early immune responses such as reactive oxygen species (ROS) bursts, nitric oxide (NO) production, hormone signaling, and MAPK activation are all tightly coupled with Ca^2+^ dynamics (Garcia Brugger et al., 2006; Bredow and Monaghan, 2019). Thus, Ca^2+^ signaling provides both specificity and versatility, serving as a pivotal regulatory hub that integrates pathogen perception with tailored adaptive responses.

Ca^2+^ signals are decoded by a set of sensor proteins, among which Calcium-Dependent Protein Kinases (CDPKs/CPKs) play a pivotal role. Unlike Calmodulin (CaM), Calmodulin-like proteins (CMLs), and Calcineurin B-like proteins (CBLs), which require downstream factors to transmit Ca^2+^ signals, CPKs uniquely combine Ca^2+^ sensing via EF-hand motifs and kinase activity within a single polypeptide (Harper et al., 2004; Boudsocq and Sheen, 2013). This dual functionality enables CPKs to directly convert Ca^2+^ fluctuations into phosphorylation cascades, activating defense gene expression and enhancing pathogen resistance (Ranty et al., 2016). This direct signaling capacity positions CPKs as crucial conduits in the transition from calcium-based perception to physiological immune outputs. Consequently, CPKs are positioned as key signaling nodes that bridge calcium dynamics with adaptive responses across plant systems.

With advances in molecular and genetic tools, our understanding of CPKs in plant immunity has increased substantially. While CPKs participate in diverse physiological processes, their functions in immunity represent one of the most dynamic and mechanistically complex aspects of Ca^2+^ signaling. Beyond their established roles as calcium sensors, recent studies indicate that CPKs often act as integrative signaling nodes that link Ca^2+^ influx to ROS production, MAPK activation, hormone signaling and transcriptional reprogramming. Framed as coordinators of multi-layered immune networks, CPKs are examined here for their role in decoding calcium signatures to regulate plant immune responses. As such, CPKs are examined here for their role as central decoders of immune-specific calcium signatures, coordinating multi-layered defense networks. Selective comparisons to other signaling contexts are included where relevant, offering insights into improving crop disease resistance while managing growth-defense trade-offs.

## 2 Structure and activation of CPKs

CPKs are pivotal signaling molecules in plants, first cloned and characterized in soybean in 1987 (Harmon et al., 1987). They are widely distributed across higher plants, green algae, oomycetes, and protists, with substantial variation in gene numbers among species. Based on phylogenetic relationships, the CPK family in terrestrial plants can be classified into four clusters (Groups I–IV) (Hamel et al., 2014). For example, 39, 34, 40, 16, and 31 members have been identified in soybean, *Arabidopsis*, maize, *Saccharina japonica*, and rice, respectively (Cheng et al., 2002; Ray et al., 2007; Kong et al., 2013; Sun et al., 2025b; Liu et al., 2016). From an evolutionary perspective, CPKs are ancient signaling molecules that emerged early in the green plant lineage and are conserved from unicellular algae to higher angiosperms (Singh et al., 2017). The modular architecture of CPKs, which consists of a kinase domain fused to a calmodulin-like domain, is thought to have evolved through gene fusion events (Schulz et al., 2013). This unique integration allows direct Ca^2+^ sensing and signal transduction within a single polypeptide (Valmonte et al., 2014). While the core structure remains conserved, lineage-specific expansion and subfunctionalization of CPK families have enabled the diversification of Ca^2+^ signaling networks across plant species, particularly in relation to immune adaptation and stress tolerance (Yip Delormel and Boudsocq, 2019).

**Figure 1:**
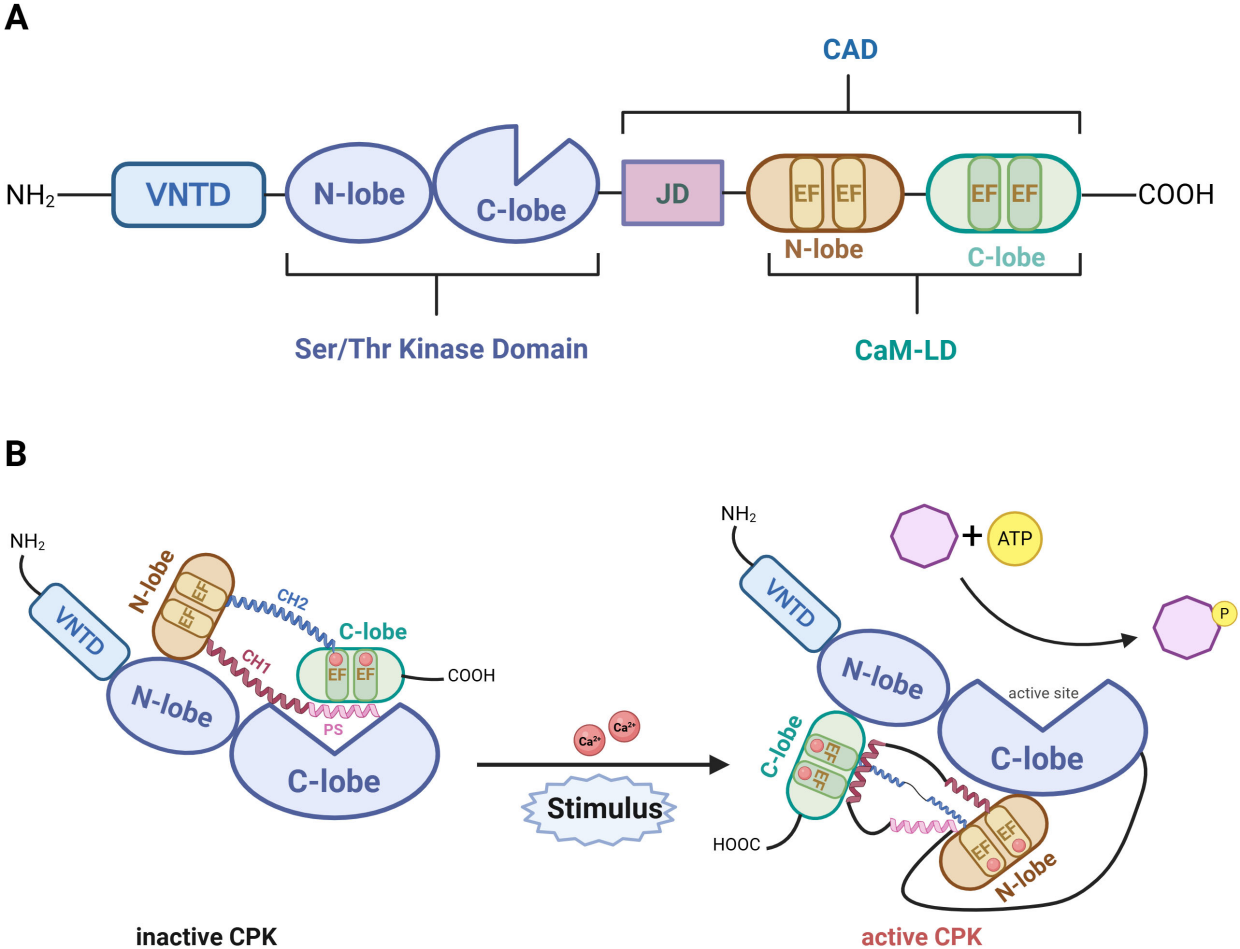
The structure of CPK. **(A)** The typical structural domains of CPKs include: the variable N-terminal domain (VNTD), serine/threonine kinase domain, and CPK activation domain (CAD). CAD is consisted by the junction domain (JD) and C-terminal calmodulin-like domain (CaM-LD). It is noteworthy that the calcium-binding and helix-loop-helix domain comprises EF-hand calcium-binding motifs which regulate CPK activity via the binding of Ca^2+^. **(B)** Inactive state: The kinase domain (KD) is autoinhibited by the junction domain (JD), which blocks the catalytic site as a pseudosubstrate. The calcium-associated domain (CAD) adopts an extended conformation. Active state: Ca^2+^ binding induces a large-scale conformational change, displacing the JD from the catalytic cleft and activating the kinase. The regulatory domain (JD-CAD) rotates ~135°, and the CAD compacts to stabilize the active conformation.

CPKs are composed of a single polypeptide chain organized into four domains: the variable N-terminal domain (VNTD), the Ser/Thr kinase domain (catalytic domain), the autoinhibitory junction domain, and the C-terminal calmodulin-like regulatory domain (CaMLD) (Klimecka and Muszyńska, 2007) (**Figure 1A**). The N-terminal variable domain is highly divergent in sequence (20–200 amino acids) and often contains lipid modifications that facilitate membrane localization. Lipidation, including myristoylation and palmitoylation, is a distinct form of post-translational modification that plays a pivotal role in modulating CPK function and localization (Willems et al., 2019). N-myristoylation and palmitoylation mediate the membrane localization of proteins and crucially regulate their substrate specificity in diverse systems (Kersten et al., 2009). Most CPKs harbor an N-myristoylation site at the second glycine and N-palmitoylation sites at the fourth or fifth cysteine residues (Cheng et al., 2002). While myristoylation is irreversible, palmitoylation is reversible, and the two modifications act together to regulate membrane association and substrate specificity (Witte et al., 2010). For instance, N-terminal myristoylation and palmitoylation facilitate the anchoring of NtCPK2 and NtCPK3 to the plasma membrane, where they undergo phosphorylation by upstream kinases and subsequently activate membrane-localized substrates involved in defense signaling (Witte et al., 2010). In this context, lipid-based modifications cooperate with phosphorylation–dephosphorylation cycles to fine-tune CPK activation thresholds and maintain the spatial precision of calcium decoding during plant stress and immune responses. The Ser/Thr kinase domain is structurally conserved and contains 11 canonical subdomains typical of protein kinases, including a conserved lysine residue in subdomain II, which functions in ATP binding (Vijayakumar et al., 2016). The autoinhibitory junction domain acts as a pseudosubstrate and is enriched in basic residues. It contains conserved regions associated with nuclear localization and maintains the kinase in an inactive state by blocking its catalytic site under low Ca^2+^ concentrations (Liese and Romeis, 2013). Upon Ca^2+^ binding, this inhibition is released, enabling kinase activation (Harmon et al., 1994; Li et al., 2019). The C-terminal CaMLD is a defining feature of CPKs, typically harboring four EF-hand calcium-binding motifs (Liu et al., 2018). These motifs occur in pairs, forming N- and C-lobes with distinct affinities for Ca^2+^, allowing sensitivity to a wide range of Ca^2+^ concentrations (Liese and Romeis, 2013).

In the resting state, the autoinhibitory junction domain occupies the catalytic site of the kinase domain, maintaining the enzyme in an inactive conformation. The autoinhibitory pseudosubstrate segment is located within the N-terminal α-helix (CH1) of the CAD. This N-terminal region interacts with the N-terminal EF lobes. A shorter second α-helix (CH2 of the CAD) connects the N-terminal EF lobe to the C-terminal EF lobe, positioned between EF-hand 2 (N-terminal) and EF-hand 3 (C-terminal). In the inactive state, the C-terminal EF-hand typically binds two calcium ions and interacts with the autoinhibitory domain, thereby contributing to structural stability. The CH1 and CH2 helices are arranged in an anti-parallel conformation and curve toward each other (Wernimont et al., 2010). Upon an increase in cytosolic Ca^2+^ concentration, Ca^2+^ binds to the EF-hand motifs, triggering conformational rearrangements in the regulatory domain. This conformational change results in the release of the autoinhibitory pseudosubstrate from the catalytic site, together with an approximate 135° reorientation of the regulatory domain relative to the kinase domain (Franz et al., 2011; Liese and Romeis, 2013). During this process, both CH1 and CH2 can break into three segments, with the autoinhibitory section of CH1 subsequently interacting with the N-EF lobe (which is bound at the C-lobe of the KD). The release of autoinhibition restores access to the catalytic site, reactivating kinase activity (**Figure 1B**). Increasing level of cellular calcium shift CPKs to conformational changes that activate phosphorylation of downstream substrates (Wernimont et al., 2010).

Although most CPKs depend on Ca^2+^ binding for activation, some isoforms such as AtCPK13 and AtCPK23 exhibit reduced Ca^2+^ dependency, a trait associated with variations in their EF-hand motifs (Kanchiswamy et al., 2010). A key insight from these studies is that several of these kinases, including AtCPK10 and AtCPK32, retain the ability to bind calcium despite their attenuated activation response (Boudsocq et al., 2012). This dissociation between calcium binding and kinase activation suggests that calcium may serve an alternative regulatory role, potentially modulating protein-protein interactions or subcellular localization rather than directly switching on the kinase (Boudsocq et al., 2012). In some cases, this low calcium dependency may stem from an inherently weak interaction between the kinase domain and the autoinhibitory domain, resulting in an unstable autoinhibitory state and conferring constitutive, calcium-independent basal activity (Li et al., 2015; Yip Delormel and Boudsocq, 2019). Consequently, the activity of these CPKs is governed by a network of alternative mechanisms. For instance, they can be integrated into phosphorylation cascades, as evidenced by calcium-activated AtCPK11 phosphorylating the calcium-insensitive AtCPK24 (Zhao et al., 2013). Furthermore, autophosphorylation events, which can modulate substrate affinity and kinase activity in a calcium-independent manner, provide an additional layer of control (Ito et al., 2017; Hu et al., 2024). Regulatory molecules such as the lipid second messenger phosphatidic acid (PA) and 14-3–3 proteins also potently enhance the activity of specific CPKs like ZmCPK11 and AtCPK21, often without altering their intrinsic calcium sensitivity (Ludwig, 2003; Klimecka and Muszyńska, 2007; Camoni et al., 1998; Van Kleeff et al., 2018). Taken together, these findings support the hypothesis that calcium-independent CPKs may represent a functionally specialized subgroup. Their intrinsic structural features, such as an unstable autoinhibitory conformation, allow them to maintain a controlled basal activity and keep the system in a primed state. Functionally, these kinases may act as rapid “triggers” that initiate early downstream responses, such as defense activation, even before full calcium transients are established. In contrast, classical calcium-sensitive CPKs serve as stimulus-specific “amplifiers” whose activities depend on the intensity and spatiotemporal dynamics of Ca^2+^ signals, thereby fine-tuning and reinforcing responses. This functional bifurcation between basal readiness and signal-dependent amplification likely represents an adaptive mechanism that enables the plant Ca^2+^ signaling network to combine rapid responsiveness with dynamic stability. Thus, CPKs function as unique Ca^2+^ sensors and responders within a single protein, integrating calcium binding with direct phosphorylation of target substrates. This dual functionality allows CPKs to rapidly decode calcium signatures and propagate signaling outputs essential for plant development and immune responses (Perochon et al., 2011).

## 3 Pathogen-triggered calcium influx as the initiator of plant immune signaling

Ca^2+^ function as universal second messengers in plants, translating external and internal stimuli into precise cellular responses. Following the structural characterization of CPKs, it becomes evident that their activation is related to cytosolic Ca^2+^ signals generated upon pathogen attack. Thus, understanding how calcium influx is initiated is essential for explaining how CPKs function as calcium decoders during immune responses.

Upon pathogen recognition, PRRs such as FLS2 and EFR perceive PAMPs on the cell surface, initiating PTI (Zhou and Zhang, 2020). This recognition activates receptor-like cytoplasmic kinases (RLCKs), including BIK1 and PBL1, which in turn phosphorylate plasma membrane calcium channels, triggering a rapid surge in cytosolic Ca^2+^ levels within seconds to minutes (Wang et al., 2024a). These kinases function as immediate signaling mediators, transmitting extracellular recognition to intracellular activation. Consequently, the activation of PRR complexes induces a series of rapid early responses, among which calcium influx is one of the most immediate and essential.

Ca^2+^ influx into the cytoplasm is mediated by multiple types of ion channels located at the plasma membrane and intracellular compartments. These include glutamate receptor-like channels (GLRs), cyclic nucleotide-gated ion channels (CNGCs), and osmotic stress-gated Ca^2+^ channels (OSCA) (Thor et al., 2020). Among them, glutamate receptor-like channels (GLRs) function as ligand-gated Ca^2+^-permeable channels that can be activated by extracellular amino acids, which accumulate during pathogen infection (Forde and Roberts, 2014). Notably, recent evidence indicates that GLRs are not exclusively localized to the plasma membrane; for instance, GLR2.9b preferentially accumulates at the nuclear envelope and contributes to Ca^2+^ signaling during ETI (Wang et al., 2025b). These findings reveal a more compartmentalized model for calcium signaling in plant immunity, where the nucleus probably acts as a semi-autonomous signaling unit. This nuclear Ca^2+^ flux potentially enables the direct and specific regulation of transcription factors and defense gene expression. Understanding this mechanism may provide new insight into the spatial control of immune signaling. Similarly, CNGCs function as non-selective Ca^2+^-permeable channels that contribute to Ca^2+^-mediated immune responses, particularly programmed cell death (Moeder et al., 2019). However, unlike their animal counterparts, recent structural evidence indicates that plant CNGCs lack a canonical cyclic nucleotide-binding domain, challenging the long-held assumption of their direct regulation by cAMP or cGMP (Wang et al., 2025a). A well-established activation mechanism in plant immunity involves phosphorylation; for example, the CNGC2 and CNGC4 complex is phosphorylated and activated by the kinase BIK1 during flg22-triggered immunity (Tian et al., 2019). This highlights phosphorylation as a primary regulatory mechanism for these channels in immunity. Future studies are needed to identify the full repertoire of kinases that regulate different CNGC isoforms and to determine how the loss of canonical ligand binding impacts the overall dynamics of calcium signaling in plant defense. Moreover, AtOSCA1.3 is phosphorylated upon flg22 perception and mediates stomatal closure during immune signaling (Thor, 2019). Recent discoveries revealed that certain NLR receptors act directly as Ca^2+^-permeable channels. The ZAR1 resistosome forms a channel complex in the plasma membrane, triggering Ca^2+^ influx and hypersensitive response (HR)-mediated cell death (Bi et al., 2021). In addition, hyperpolarization-activated calcium channels (HACCs) are activated by ROS following transient [Ca^2+^]_cyt_ elevations, further shaping feedback regulation (Park and Shin, 2022). Thus, plant immune-related Ca^2+^ signaling involves a coordinated network of channels with diverse subcellular localizations and activation mechanisms. Future studies are needed to elucidate how these diverse channels are spatially and temporally coordinated to shape the precise calcium signatures that govern immunity.

Organelles play essential roles in maintaining cytosolic Ca^2+^ homeostasis. Both influx and efflux of Ca^2+^ from organelles, such as chloroplasts, vacuoles, and the nucleus, contribute to the overall Ca^2+^ signaling network. Notably, nuclear Ca^2+^ fluctuations directly affect transcriptional reprogramming during immune activation (Wang et al., 2024b), highlighting the critical role of subcellular calcium partitioning in defense signaling. Beyond the nucleus, the plant cytoskeleton serves as both a sensor and transducer of calcium signals. Cytoskeletal components, including microfilaments (MFs) and microtubules (MTs), can bind Ca^2+^ directly or indirectly and are dynamically remodeled upon pathogen perception (Wang et al., 2022). During PTI, MFs reorganize in response to diverse pathogens through the action of actin-binding proteins (ABPs) (Tian et al., 2009), suggesting that the cytoskeleton acts as a conserved structural platform for early defense. This remodeling capacity is particularly crucial during ETI, as exemplified by the finding that in AvrPphB-induced immunity, CPK3 phosphorylates actin depolymerizing factor 4 (ADF4). This phosphorylation reduces ADF4’s affinity for actin filaments, promotes MF bundling, and ultimately activates immune responses (Lu et al., 2020).

The spatiotemporal patterns of Ca^2+^ signals are decoded by CPK, which propagate and amplify the signal to regulate transcriptional reprogramming and defense gene expression (Lee and Seo, 2021; Ranty et al., 2016). Specifically, when cytosolic Ca^2+^ concentrations rise, Ca^2+^ binds to the EF-hand motifs within the calmodulin-like domain of CPKs, inducing a conformational change that releases the autoinhibitory junction from the kinase catalytic domain. This structural rearrangement activates the kinase, enabling it to phosphorylate downstream substrates such as RBOHD and transcription factors, thereby coupling ionic signals to immune outputs (Schulz et al., 2013; Yip Delormel and Boudsocq, 2019; Luan and Wang, 2021). For example, activated CPK5 directly phosphorylates and activates RBOHD, initiating the production of reactive oxygen species (ROS) (Guerra et al., 2020). The resulting ROS further promotes calcium influx, thereby establishing a self-reinforcing Ca^2+^/ROS positive feedback loop. This circuit not only amplifies local defense responses but also facilitates the propagation of defense signals from the infection site to distal tissues, providing a rapid immune activation basis for PTI, ETI, and systemic acquired resistance (SAR) (Dubiella et al., 2013; Köster et al., 2025). Differential expression of CPK across tissues and time scales ensures specificity of calcium-mediated responses. For instance, stomatal defense responses are regulated by Ca^2+^ signaling cascades that control guard cell dynamics (Arnaud and Hwang, 2015). CPKs serve as central sensors in this pathway, converting Ca^2+^ influx into immune outputs. In addition to local signaling, systemic propagation of immune cues has also been observed. For example, the bacterial elicitor flg22 can travel through vascular connections to distal tissues without accumulating itself, suggesting that Ca^2+^ transients may serve as the actual mobile signals that initiate defense responses at distant sites (Jelenska et al., 2017).

Following the decoding of calcium signals by sensors such as CPKs, restoration of cellular calcium homeostasis is crucial for terminating the signal and preventing cytotoxicity. Ca^2+^ efflux is mediated by Ca^2+^-ATPase pumps and Ca^2+^/cation antiporters, which restore basal [Ca^2+^]_cyt_ levels (Jiang and Ding, 2023). The precise orchestration of this homeostasis network is essential for coordinating plant immunity with normal growth processes (Wang and Luan, 2024; Wang et al., 2024a). Autoinhibited Ca^2+^-ATPases (ACAs), belonging to the P-type Ca^2+^-ATPase family, export cytosolic Ca^2+^ using ATP hydrolysis (Park and Shin, 2022). The Ca^2+^/cation antiporter (CaCA) superfamily also plays a crucial role in cation homeostasis and stress responses. In plants, CaCA proteins are classified into four groups: H^+^/cation exchangers (CAXs), cation/Ca^2+^ exchangers (CCXs), Mg^2+^/H^+^ exchangers (MHXs), and Na^+^/Ca^2+^ exchanger-like proteins (NCLs) (Taneja et al., 2016; Amagaya et al., 2019). It is tempting to speculate that the calcium sensors themselves, such as specific CPKs or CBL-CIPK complexes, might directly phosphorylate and activate these efflux proteins once a particular threshold or duration of calcium elevation is reached. A key challenge will be to determine the precise triggers that activate scavenging transporters to terminate signaling.

The activation and formation of specific calcium signatures serve as the fundamental prerequisite for CPK-mediated immune responses in plants. In this case, it will be interesting to address how distinct Ca^2+^ signatures selectively activate specific CPK isoforms to fine-tune immune outcomes. Precise analysis of calcium dynamics and CPK activation kinetics will help to clarify this relationship. Future research should focus on elucidating how spatiotemporal coordination among diverse calcium channels shapes these immune-specific calcium signatures, and uncovering the molecular links between calcium signaling termination and the appropriate resolution of immune responses.

## 4 CPKs as central signaling hubs integrating PTI and ETI

Plants have evolved complex signaling networks to cope with continuous environmental fluctuations and the constant threat of pathogen invasion (Schneider, 2002). CPKs have emerged as central integrators of plant immune signaling, functioning as critical convergence points for both PTI and ETI. The unique structural architecture of CPKs, which integrates calcium-sensing and kinase functionalities within a single protein molecule, enables their direct participation in decoding pathogen-induced calcium signatures and initiating appropriate defense responses. This integrated architecture positions CPKs as essential components in the plant’s immune network, allowing for rapid signal transduction from pathogen perception to defense activation.

During PTI, CPKs are rapidly activated following pattern recognition receptor (PRR) complex assembly. The initial immune response involves activated BIK1, which phosphorylates the Ca^2+^ channels CNGC2/4 and the NADPH oxidase RBOHD, thereby mediating calcium entry and ROS production (Tian et al., 2019). CPKs act immediately downstream of this process to reinforce and fine-tune signaling. Importantly, subgroup I CPKs, including AtCPK4/5/6/11, then amplify this signal through direct phosphorylation of RBOHD, enhancing oxidative bursts that characterize early PTI signaling (Dubiella et al., 2013). Concurrently, negative regulators like AtCPK28 provide essential feedback control by phosphorylating PUB25 and PUB26, thereby modulating BIK1 accumulation and fine-tuning receptor-proximal signaling strength (Monaghan et al., 2015). These findings strongly suggest that CPKs function not merely as signal transducers but as sophisticated managers of PTI intensity, balancing rapid activation with appropriate constraint to prevent excessive immune responses. Moreover, CPK cross-regulates PTI signaling with the mitogen-activated protein kinase (MAPK) pathway.

In contrast, ETI triggers stronger and more sustained cytosolic Ca^2+^ increase, driving prolonged CPK activation (Bi et al., 2021; Wang et al., 2024b). This temporal distinction in calcium signatures ensures differential activation of specific CPK isoforms, directing them toward more pronounced defense outcomes such as hypersensitive response (Dodds and Rathjen, 2010). Notably, isoforms like AtCPK3 participate in both PTI and ETI contexts and mediate programmed cell death, illustrating the functional versatility of CPKs in adapting to different immune challenges (Lu et al., 2020; Lachaud et al., 2013). These results indicate that individual CPK isoforms can be recruited to distinct immune programs based on the nature of the calcium signature they encounter. However, the precise mechanism governing this isoform-specific recruitment remains unclear and deserves further investigation. This functional overlap extends to shared signaling modules, including MAPK cascades, hormone pathways, and calcium signaling, which converge to activate defense gene expression and localized cell death (Zhang et al., 2007; Postel and Kemmerling, 2009; Dodds and Rathjen, 2010; Yu et al., 2017; Kimura et al., 2020).

Recent advances have revealed that PTI and ETI are not independent but are mutually reinforcing processes, each enhancing the other to produce robust plant immunity (Ngou et al., 2021; Yuan et al., 2021). CPKs function as pivotal integrators of both PTI and ETI by decoding the distinct calcium signatures associated with each layer and coupling receptor activation to downstream phosphorylation cascades. OsCPK5 and OsCPK13 act as positive regulators of PTI against rice blast fungus, but their simultaneous loss triggers NLR (OsCARP1)-dependent ETI, leading to enhanced resistance (Wang et al., 2025c). CPKs maintain a delicate balance between different immune layers. CPK4/5/6/11 translocates to the nucleus and phosphorylates the transcription factors WRKY8/28/48, which collaboratively regulate transcriptional reprogramming essential for NLR-dependent pathogen growth restriction. Furthermore, CPK activity is dynamically regulated at the subcellular level. AtCPK16 relocates from the plasma membrane to chloroplasts after flg22 perception, where it contributes to chloroplast-mediated defenses and promotes salicylic acid biosynthesis (Medina Puche et al., 2020). These spatial dynamics significantly enhance the scope of CPK-mediated immune regulation, thus revealing an additional layer of complexity in plant immune signaling organization.

The complex spatiotemporal regulation of CPKs enables them to execute a diverse functional repertoire, which includes both positive and negative regulatory roles to ensure immune homeostasis. Recent genetic and biochemical evidence establishes that CPK3 and functionally related family members directly phosphorylate the TIR domain of NLRs, inhibiting NADase activity and constraining excessive immunity (Li et al., 2025). This finding reveals an additional layer of CPK-mediated control at the core of ETI signaling. Similar regulatory roles exist in monocots. OsCPK4, a rice subgroup II CPK, phosphorylates OsRLCK176 (the ortholog of AtBIK1), leading to its degradation and attenuation of immune signaling (Wang et al., 2018). Interestingly, another study has shown that overexpression of *OsCPK4* enhances rice resistance to fungal diseases (Bundó and Coca, 2016), which may be due to different phosphorylation states of OsCPK4 (Wang et al., 2018). Since the overexpression of *OsCPK4* also confers salt and drought tolerance to plants (Campo et al., 2014), *OsCPK4* is also a convergence point for the positive regulation of biotic and abiotic stress in rice. Similarly, OsCPK12 enhances salt tolerance, yet its overexpression compromises resistance to rice blast, exemplifying the trade-offs between abiotic and biotic stress regulation (Asano et al., 2012; Li et al., 2020). These findings reveal that CPK-mediated stress adaptation operates through complex regulatory networks where individual isoforms can function as either synergistic coordinators or antagonistic regulators of different stress pathways, depending on cellular context and environmental conditions. The OsCPK17-OsPUB12 module further refines this regulation by maintaining OsRLCK176 homeostasis, representing a finely balanced regulatory circuit (Mou et al., 2024). In this case, it will be interesting to address how these opposing regulatory activities are coordinated to maintain immune homeostasis without compromising defense capacity. *OsCPK12* (a member of subgroup II CPKs) has been identified as a negative regulator of defense. By impairing ROS generation and increasing ABA sensitivity, OsCPK12 compromises resistance to blast pathogens, highlighting its role in balancing growth and immunity in a key crop species (Asano et al., 2012). Under heat stress, activated OsCPK24/28 phosphorylates OsHSFA4d, which in turn transcriptionally upregulates CslF6 (Fang et al., 2025). This induction directly suppresses PAMP-triggered ROS bursts and defense gene expression, illustrating a direct molecular pathway through which thermotolerance signaling negatively regulates plant immunity.

The intricate balance between positive and negative regulation by CPKs gives rise to their remarkable functional diversity, which can be understood along two major dimensions: redundancy and lineage-specific specialization. Redundancy ensures robust signaling, as seen in the cooperative roles of AtCPK4/5/6/11 during flg22-triggered resistance to *Pseudomonas syringae* (Romeis and Herde, 2014). Lineage-specific specialization highlights how CPK repertoires evolve to meet species-specific immune challenges (**Table 1**). In addition to these functions, multiple lines of genetic and biochemical evidence indicate that CPKs broadly participate in regulating plant resistance to pathogens and can strengthen host immunity by modulating diverse signaling processes (Luo and Reidy, 2002). By engaging in functional interactions with other signaling molecules, CPKs assemble complex regulatory networks that coordinate both local and systemic responses. CPK decodes calcium signals to activate specific local defense responses, while also promoting distal transcriptional reprogramming and pathogen resistance throughout the plant via the propagation of defense signals between cells (Romeis and Herde, 2014). This capacity for spatial coordination underscores the centrality of CPKs in establishing comprehensive immune protection throughout the plant. Nonetheless, the mechanisms underlying CPK-mediated systemic signaling have not been thoroughly investigated and represent a promising area for future research.

**Table 1:** A comprehensive summary of CPK functions in plant immunity.

Species/Gene	Functional role	Function summary	References
*Arabidopsis thaliana*			
AtCPK1	Positive Regulator	Activates SA biosynthetic genes (PAD4, SID2) and SA-responsive genes, enhancing broad-spectrum resistance.	(Coca and San Segundo, 2010)
AtCPK3	Positive Regulator	Regulates both PTI and ETI; required for sphingolipid-induced PCD.	(Lu et al., 2020; Lachaud et al., 2013)
AtCPK4/5/6/11	Positive Regulators	Function redundantly in flg22 signaling; phosphorylate RBOHD to trigger ROS burst and WRKY28/48 to activate defense genes.	(Romeis and Herde, 2014; Dubiella et al., 2013)
AtCPK5	Positive Regulator	Overexpression enhances powdery mildew resistance; interacts with TN2 to degrade CAMTA3, regulating autoimmunity.	(Liu et al., 2017, 2024)
AtCPK5/6	Positive Regulators	Mediate immune response to *Botrytis cinerea* through regulation of ethylene biosynthesis.	(Gravino et al., 2015)
AtCPK16	Positive Regulator	Relocates to chloroplasts upon elicitation, regulating chloroplast defenses and SA biosynthesis.	(Medina Puche et al., 2020)
AtCPK28	Negative Regulator	Phosphorylates PUB25/26 to maintain BIK1 homeostasis; loss-of-function enhances resistance.	(Kadota et al., 2014; Monaghan et al., 2015)
*Oryza sativa*			
OsCPK4	Dual Regulator	Phosphorylates OsRLCK176 for degradation (negative); enhances fungal resistance under certain conditions and confers abiotic stress tolerance (positive).	(Wang et al., 2018; Bundó and Coca, 2017; Campo et al., 2014)
OsCPK5	Positive Regulator	Phosphorylates OsERG1 for PM targeting, converting Ca^2+^ signals into defense.	(Kang et al., 2013)
OsCPK10	Positive Regulator	Activates CAT-A to scavenge H_2_O_2_, mediating blast resistance.	(Bundó and Coca, 2017)
*OsCPK12*	Dual Regulator	Overexpression increases blast susceptibility (negative); positively regulates salt tolerance.	(Asano et al., 2012; Li et al., 2020)
OsCPK17	Positive Regulator	Maintains OsRLCK176 homeostasis with OsPUB12.	(Mou et al., 2024)
OsCPK4/18	Negative Regulator	phosphorylate OsMPK5 at two conserved threonine residues, which suppresses defense gene expression while promoting growth-related pathways.	(Xie et al., 2014; Li et al., 2022).
*Nicotiana tabacum*			
*NtCPK2*	Positive Regulator	Stimulates JA and ethylene accumulation, inducing downstream defense genes.	(Ludwig et al., 2005)
*Hordeum vulgare*			
*HvCPK3*	Negative Regulator	Facilitates penetration by *Blumeria graminis* in *mlo*-resistant plants.	(Freymark et al., 2007)
*HvCPK4*	Positive Regulator	Autoinhibitory junction (J4) functions as dominant-negative, confirming its positive role in defense.	(Freymark et al., 2007)
*Triticum aestivum*			
*TaCPK2*	Positive Regulator	Essential for resistance to *Blumeria graminis*; enhances resistance in rice via JA/SA signaling.	(Geng et al., 2013)
*TaCPK7*	Positive Regulator	Enhances resistance to stripe rust (*Puccinia striiformis*) by promoting ROS burst and induction of PR genes.	(Goher et al., 2024)
*TaCPK27*	Negative Regulator	Acts as a susceptibility factor by suppressing autophagy and promoting PCD (ROS dependent), leading to compromised resistance to powdery mildew.	(Yue et al., 2023)
*Musa acuminate*			
*MaCPK2/4*	Positive Regulators	Confer resistance against *Fusarium oxysporum* (Foc TR4).	(Wang et al., 2016b)
*Capsicum annuum*			
*CaCPK15*	Positive Regulator	Silencing increases susceptibility to *Ralstonia solanacearum*; downregulates CaPR1 and CaNPR1; Triggers hypersensitive cell death (HR) and H_2_O_2_ accumulation; forms a positive feedback loop with CaWRKY40 to amplify defense signaling.	(Shen et al., 2016)
*Litchi chinensis*			
*LcCPK5/17/19*	Putative Positive Regulators	Upregulated after pathogen treatment; potentially function in SA pathway.	(Liu et al., 2023)
LcCPK8	Putative Negative Regulator	May negatively regulate pathogen stress response.	(Liu et al., 2023)
*Solanum lycopersicum*			
SlCPK10/18	Positive Regulators	Contribute to non-host and host resistance against *Pseudomonas syringae*.	(Wang et al., 2016a)
*Solanum tuberosum*			
*StCPK4/5*	Positive Regulators	Regulate early plant defense by controlling StRBOHB-dependent ROS production and subsequent defense gene expression.	(Kobayashi et al., 2007)
*StCPK5*	Dual Regulator	Confers resistance to *P. infestans* but simultaneously reduces resistance to late blight, highlighting pathway trade-offs.	(Kobayashi et al., 2012)
StCPK7	Positive Regulator	Phosphorylates the key defense enzyme Phenylalanine ammonia-lyase (PAL) to enhance resistance against P. infestans.	(Fantino et al., 2017)
*Manihot esculenta*			
MeCPK1	Positive Regulator	Interacts with MeHSP90.9 to activate immune responses via MeSRS1 and MeWRKY20.	(Wei et al., 2024)
*Hami Melon*			
*HmCPK2*	Positive Regulator	Expression enhanced by Ca^2+^ priming, contributing to *Penicillium* resistance.	(Ning et al., 2019)
*Vitis pseudoreticulata*			
VpCPK9, VpCPK13	Positive Regulator	Interact with and phosphorylate ethylene biosynthetic enzymesVpACS1/2, integrating SA and ethylene signaling for enhanced powdery mildew resistance.	(Hu et al., 2021)
*Zea mays*			
*ZmCPK10*	Positive Regulator	Ca^2+^ -responsive kinase whose expression is induced during seedling development and in response to fungal infection and elicitor treatments.	(Murillo et al., 2001)
ZmCPK39	Negative Regulator	phosphorylating and degrading the transcription factor ZmDi19, which in turn represses the expression of the defense gene ZmPR10	(Zhu et al., 2024)

In summary, CPKs function as master regulators that interpret calcium dynamics to coordinate appropriately scaled defense responses across PTI and ETI. Their activities encompass direct activation of oxidative bursts, transcriptional reprogramming, feedback-mediated homeostasis, and lineage-specific adaptations. The next challenge will be to demonstrate how specific CPK isoforms integrate multiple signaling cues to execute context-appropriate immune responses while maintaining growth-defense balance. Understanding the underlying mechanism may provide new insight into the plant immune system and facilitate the development of novel strategies for crop improvement.

## 5 CPK–MAPK crosstalk: converging pathways in plant immune signaling

CPKs and MAPKs represent two central kinase modules that decode pathogen-induced signals into tailored immune responses (Boudsocq et al., 2010). Although each pathway can independently regulate defense, accumulating evidence indicates extensive crosstalk between them, allowing plants to integrate calcium signals and phosphorylation cascades into coordinated outputs. This functional intersection not only amplifies immune signaling but also provides specificity to pathogen-responsive transcriptional programs. A canonical MAPK cascade is composed of at least one MAPK (MPK), one MAPK kinase (MAPKK or MKK), and one MAPKK kinase (MAPKKK or MEKK) (Zhang and Zhang, 2022). Through sequential phosphorylation, MAPK cascades transmit and amplify both external and internal cues, ultimately regulating the expression of resistance genes (Hamel et al., 2006). Importantly, CPKs intersect with these cascades at multiple levels, often sharing substrates with MAPKs or directly influencing MAPK activity.

The same transcription factors can perform different functions in plant PTI and ETI signal transduction or in response to various pathogens. In *Arabidopsis*, both CPK5/6 and MPK3/6 are capable of phosphorylating WRKY33 and this dual regulation synergistically enhances camalexin biosynthesis, a critical phytoalexin in defense against pathogens (Zhou et al., 2020). Extensive research has established the transcription factor WRKY33 as a major point of convergence. This example illustrates how CPK- and MAPK-dependent signaling can converge on common transcriptional regulators to fine-tune metabolic outputs. Additional evidence further supports this cooperative role. Four CPKs in *Arabidopsis* (CPK4/5/6/11) have been identified as playing a crucial role in relaying primary MAMP immune signals in conjunction with the MAPK cascade (Boudsocq et al., 2010). The *cpk5/cpk6*, cpk*5/cpk6/cpk11*, and cpk*4/cpk5/cpk6/cpk11* mutants impair the flg22-induced response, including the burst of ROS (Boudsocq et al., 2010). Constitutively active variants of these CPKs phosphorylate WRKY8, WRKY28, and WRKY48, driving transcriptional reprogramming, while these WRKYs themselves are also transcriptionally modulated by stress conditions (Gao et al., 2013; Jiang et al., 2017).

Crosstalk is also evident in monocots. In rice, OsMPK5 is activated by both biotic and abiotic stresses. Earlier work established that OsCPK4 and OsCPK18 phosphorylate OsMPK5 at two conserved threonine residues (Thr-14 and Thr-32), which suppresses defense gene expression while promoting growth-related pathways (Xie et al., 2014; Li et al., 2022). A more recent study has expanded this model, demonstrating that OsCPK18-activated OsMPK5 in turn negatively regulates rice defense against *M. oryzae* by phosphorylating substrates such as OsDRB1.4 (Chen et al., 2025). Similarly, OsCPK5 and OsCPK13 directly phosphorylate the canonical TXY activation motif of OsMPK3/6 under salt stress, thereby activating these MAPKs independently of upstream MKKs to enhance tolerance (Su et al., 2024). This demonstrates that CPK-MAPK modules can function as either inhibitory or activating switches, fine-tuning plant responses to diverse environmental cues. Beyond phosphorylation, CPKs may also act as scaffolds that modulate MAPK signaling independent of their kinase activity. In grapevine (*Vitis vinifera L.*), VpCPK9 and VpCPK13 physically associate with VpMAPK3 and VpMAPK6, altering their phosphorylation status and protein stability (Hu et al., 2021). Remarkably, even kinase-dead variants of these CPKs retained this ability, underscoring a scaffolding role that ensures signaling specificity and efficiency within immune networks (Hu et al., 2021). Such non-catalytic functions further expand the versatility of CPKs in shaping MAPK-dependent responses. This non-catalytic function reveals an additional layer of complexity in CPK-MAPK interactions, where CPKs serve as platform organizers rather than merely enzymatic activators.

Together, these findings establish CPK–MAPK crosstalk as a pivotal mechanism that integrates calcium-dependent and phosphorylation-based signaling. By converging on shared substrates, directly modifying MAPK activity, or functioning as scaffolds, CPKs provide an additional layer of regulation that strengthens and refines plant immunity. This intersection highlights how plants exploit modular kinase networks to balance growth, stress adaptation, and disease resistance. Future research should focus on elucidating the precise phosphorylation codes that govern CPK-MAPK communication, and how these cross-talk modules are spatially organized within cells to ensure signaling specificity. Understanding how plants rewire these kinase networks under different environmental scenarios will be crucial for developing crops with enhanced resilience.

## 6 CPKs as integrators of ROS, hypersensitive response, and hormone signaling

Beyond their coordination with MAPK cascades, CPKs play an equally crucial role in regulating reactive oxygen species (ROS) signaling, a cornerstone of plant immunity. Upon pathogen challenge, CPK activity rises rapidly, initiating a HR characterized by localized programmed cell death (PCD) that restricts pathogen spread. A central mechanism involves the phosphorylation of plasma membrane NADPH oxidases (RBOHs), which catalyze ROS production, a hallmark of early defense responses (Yip Delormel and Boudsocq, 2019). These findings strongly suggest that CPK-mediated phosphorylation serves as a critical switch for activating the oxidative burst. Functional specialization among CPK isoforms is evident from their differential impact on defense hormone pathways. For instance, overexpression of *AtCPK1* in *Arabidopsis* triggers SA accumulation and the expression of SA-dependent defense genes, thereby conferring broad-spectrum resistance to fungal (*Fusarium oxysporum*, *Botrytis cinerea*) and bacterial (*Pseudomonas syringae*) pathogens (Lee and Rudd, 2002; Coca and San Segundo, 2010). Similarly, constitutively active *NtCPK2* in tobacco stimulates JA and ethylene biosynthesis, inducing downstream defense genes (Ludwig et al., 2005). These findings illustrate the functional divergence of CPK isoforms in regulating defense pathways.

ROS bursts in immunity are mediated primarily by RBOHD and RBOHF (Suzuki et al., 2011). Importantly, compared with PTI, ETI induces more sustained Ca^2+^ influx, which prolongs ROS production and amplifies oxidative stress (Simeunovic et al., 2016). Moreover, Ca^2+^ not only regulates RBOHs through EF-hand motifs but also promotes their phosphorylation by CPKs, which together contribute to ROS generation and PCD (Kobayashi et al., 2007; Gao et al., 2013; Dubiella et al., 2013). The diffusibility of H_2_O_2_ enables the transmission of immune signals across cells, linking local perception of pathogens to systemic defense activation (Dubiella et al., 2013; Romeis and Herde, 2014). Surprisingly, Upon recognition of *AvrRpm1*, CPK12 is Ca^2+^-dependently activated and phosphorylates the plasma membrane aquaporin PIP2;1 at S280/S283, facilitating apoplastic ROS import into the cytoplasm and enhancing resistance against *Pst DC3000* (*avrRpm1*). This process is independent of RBOHD phosphorylation, highlighting functional specialization among CPK members in ETI signaling.

In parallel to ROS control, CPKs extensively interface with defense-related hormone signaling pathways to ensure appropriate response coordination. Salicylic acid (SA) and jasmonic acid (JA) are pivotal defense hormones that mediate resistance to biotrophic and hemi-biotrophic pathogens (Geng et al., 2013). In *Arabidopsis*, *AtCPK1* activates *PAD4* and *SID2*/*ICS1*, enhancing SA biosynthesis and SA-responsive gene expression while leaving JA and ethylene pathways largely unaffected (Coca and San Segundo, 2010). However, AtCPK5 and AtCPK6 regulate *ACS* gene expression during *Botrytis cinerea* infection, thereby tuning ethylene biosynthesis (Gravino et al., 2015). Notably, CPK5 employs multiple strategies to enhance immunity, including interacting with TN2 to degrade CAMTA3 and phosphorylating MORC1 to promote its nuclear import and stability, thereby enabling broad-spectrum resistance (Liu et al., 2024; Sun et al., 2025a). These results highlight how individual CPK isoforms have evolved specialized mechanisms to fine-tune defense signaling, with some maintaining pathway specificity while others integrate multiple regulatory layers to achieve broad-spectrum resistance. Similarly,In rice, OsCPK10 boosts rice blast resistance by activating CAT-A to scavenge H_2_O_2_, thereby mediating blast disease resistance (Bundó and Coca, 2017). Moreover, constitutively active *OsCPK10* elevates both SA- and JA-related defense genes and enhances resistance to *Pseudomonas syringae* pv. *tomato* (Fu et al., 2013). These findings indicate that OsCPK10 can coordinately activate multiple hormone pathways for broad-spectrum defense. *TaCPK2-A* is essential for wheat resistance to *Blumeria graminis tritici* (Bgt) (Geng et al., 2013). Heterologous expression of *TaCPK2-A* in rice enhances *Xanthomonas oryzae* pv. *Oryzae* (Xoo) resistance by upregulating WRKY45–1 expression and activating JA/SA-mediated signaling (Geng et al., 2013). Together, these studies demonstrate that CPKs convert calcium signatures into coordinated ROS and hormone responses tailored to pathogen lifestyle.

Pathogens have also evolved strategies to manipulate CPK-mediated signaling. For instance, alternaric acid (AA), a major toxin produced by *Alternaria solani*, inhibits hypersensitive cell death by stimulating host CPK activity, thereby suppressing HR (Furuichi, 2019). Conversely, in the tomato *Cf-9*/*Avr9* system, effector recognition induces CPK expression; silencing CPKs abolishes the *Cf*-*Avr9*-elicited HR in *Nicotiana benthamiana*, indicating that CPK activity is indispensable for this ETI response (Romeis, 2001). In pepper, overexpression of *CaCPK15* triggers hypersensitive cell death and H_2_O_2_ accumulation and indirectly upregulates CaWRKY40, which binds the *CaCPK15* promoter to establish a positive feedback loop that amplifies CPK-mediated defense signaling (Shen et al., 2016). These contrasting examples illustrate that CPKs are both targets of pathogen interference and essential mediators of effector-triggered defense. Furthermore, the functional repertoire of CPKs is marked by considerable complexity across the plant kingdom, with extensive evidence from diverse crop species revealing highly isoform-specific and context-dependent roles in defense and pathogen manipulation (**Table 1**).

**Figure 2:**
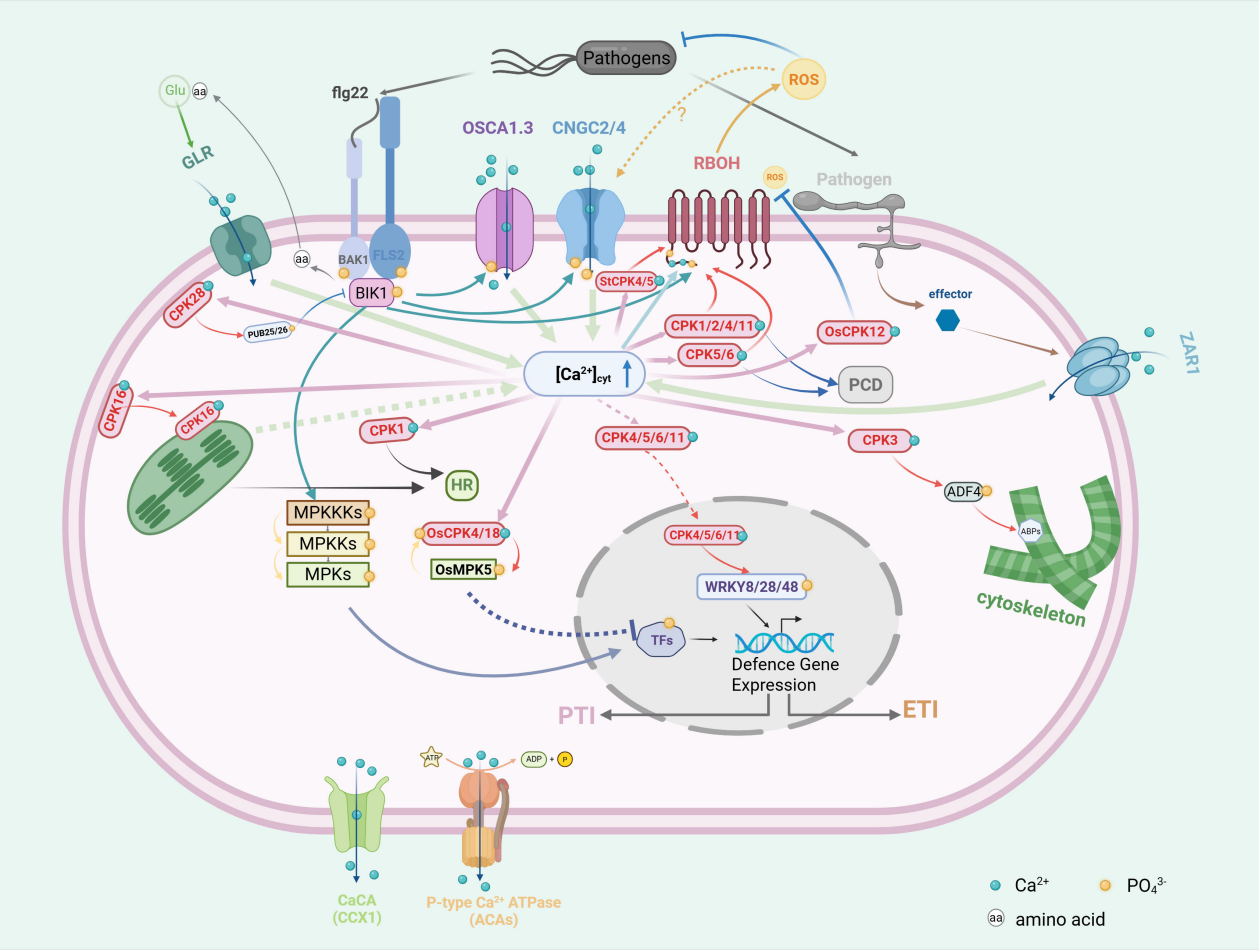
Signaling pathway of CPK in plant disease resistance. Pattern recognition receptors (PRRs) on the plant plasma membrane detect the bacterial flagellin epitope flg22, leading to the activation of BIK1, which subsequently dissociates from the FLS2-BAK1 complex. Phosphorylated BIK1 further triggers calcium influx by phosphorylating CNGC2/4 and OSCA1.3. ZAR1, activated by pathogen effectors, mediates calcium ion influx, and the resulting calcium signals are decoded by CPKs. Activated CPKs, in concert with BIK1, activate RBOH, which generates reactive oxygen species (ROS) that, in turn, stimulate CNGC2/4, sustaining an increase in cytosolic calcium ion concentration. CPK28 promotes the ubiquitination of PUB25/26, thereby controlling BIK1 accumulation. CPK16, upon activation on the plasma membrane, translocates to chloroplasts, enhancing immune responses within the organelle. CPK3 strengthens the cytoskeleton by phosphorylating ADF4, thereby activating immune responses. BIK1 initiates a mitogen-activated protein kinase (MPK) phosphorylation cascade, with MPK5 interacting with OsCPK4/18 to inhibit downstream transcription factors. CPK4/5/6/11 phosphorylate transcription factors WRKY8/28/48, activating the expression of downstream defense genes and triggering both PTI and ETI immune responses.

Taken together, these findings establish CPKs as central regulators of HR, ROS bursts, and hormone-mediated signaling in plant immunity. To synthesize these insights, a conceptual model was constructed to summarize the central role of CPKs in plant immunity. The model depicts sequential events from pathogen recognition and Ca^2+^ influx to CPK activation and downstream signaling. CPKs function as core decoders of Ca^2+^ transients, phosphorylating diverse substrates to trigger ROS production, activate MAPK cascades, regulate hormone signaling and transcriptional reprogramming. Collectively, this framework underscores how CPKs integrate multiple defense pathways to coordinate both local and systemic immune responses (**Figure 2**). Beyond these local regulatory roles, Ca^2+^ balance itself has emerged as a key determinant of growth–defense trade-offs. Recent findings demonstrate that Ca^2+^ homeostasis can function as a molecular switch, coordinating immune activation with developmental processes to prevent excessive costs to growth (Wang et al., 2024a). This observation suggests that CPKs, as central Ca^2+^ decoders, may also participate in fine-tuning this balance between resistance and growth. The multifunctional nature of CPKs underscores their significance as promising targets for engineering durable and broad-spectrum disease resistance in crops. The emerging challenge is to establish precise functional identities for each isoform and define the molecular contexts that determine their specific contributions to plant immunity.

## 7 Discussion

During long-term evolution, plants have developed sophisticated and multilayered regulatory mechanisms to counter pathogen invasion and limit tissue damage. These defense strategies involve changes at the morphological, physiological, biochemical, and molecular levels, forming a complex regulatory network to modulate the transduction of disease resistance signals and cellular homeostasis. The complexity of plant disease resistance responses is reflected in their interactions with pathogens, which involve numerous regulatory factors and genes. Therefore, a deep understanding of how plants prevent and control diseases is of vital importance for enhancing agricultural productivity and protecting the ecological environment. Substantial evidence now establishes CPKs as central integrators that connect calcium signaling with ROS production, hormone pathways, and MAPK cascades to coordinate plant immune responses. However, our understanding of how CPK-mediated signaling achieves both specificity and flexibility remains fragmented.

Calcium-dependent phosphorylation represents a central regulatory mechanism in plant immunity. Because individual CPK isoforms exhibit distinct calcium sensitivities, their activation cannot be explained solely by calcium binding. In addition to Ca^2+^-dependent activation, an important regulatory process involves intramolecular autophosphorylation, which adjusts the activation threshold of specific CPKs and modulates their signaling output. CPK28 provides a well-documented example: it undergoes intermolecular autophosphorylation at a conserved Ser318 residue, which lowers its activation threshold without altering substrate specificity (Bredow et al., 2021) This priming mechanism enables CPK28 to maintain basal activity at resting cellular Ca^2+^ levels, which is essential for its function in immune homeostasis through BIK1 degradation. While the regulatory role of CPK28 in plant immunity has been relatively comprehensively studied, the mechanisms of other CPKs remain to be further elucidated. A key objective for future research is to solve the full crystal structures of CPKs, which will provide insights into the structural role of phosphorylation and the activation mechanism of these kinases. Regulation also occurs between different kinase family members. AtCIPK26 and AtCPK5 were shown to engage in reciprocal trans-phosphorylation, which strengthens their auto-phosphorylation and generates a synergistically activated kinase pair (Köster et al., 2025). This super-activated module generates a two-layer phospho-code, which enhances phosphorylation of common RBOHD sites and activates key regulatory residues to enhance RBOHD responsiveness to ambient Ca^2+^. Together, these studies demonstrate that phosphorylation modulates CPK activity at multiple levels, enabling plants to translate subtle calcium fluctuations into precise immune responses. We hypothesize that an elegant mechanism may exist whereby low-amplitude calcium waves potently activate ROS production for systemic signaling, without necessarily triggering deleterious responses in bystander cells. Clarifying the composition and dynamics of these modules will help establish how plants achieve specificity in calcium-dependent immune signaling.

Balancing growth and immunity represent a central challenge for plants, as strong defense activation often imposes measurable fitness costs. Natural variation studies showing a negative correlation between NLR gene density and growth potential support this trade-off (Giolai and Laine, 2024) These findings indicate that plants require mechanisms to modulate immune intensity according to development and environmental conditions. CPKs contribute to this balance by functioning as calcium-dependent integrators that adjust signaling priorities in a context-specific manner. One way in which CPKs regulate this balance is by modulating defense gene expression when growth demands are high. In rice, the OsCPK18–OsMPK5–OsDRB1.4 module suppresses defense gene expression at the post-transcriptional level to optimize energy allocation during stress (Chen et al., 2025). Beyond resource allocation, CPKs enable dynamic priority shifts in response to changing environmental conditions. Under heat stress, the OsCPK24/28–OsHSFA4d pathway promotes thermotolerance through HSP101 induction but transiently reduces immune capacity (Fang et al., 2025). This capacity enables CPKs to dynamically adjust defense investment based on the predominant stress, thereby optimizing plant fitness under fluctuating conditions. Given that localized Ca^2+^ signatures are known to differ across tissues, it’s likely that similar priority adjustments occur in other tissues, where CPK activation may depend on localized calcium elevations generated by temperature or osmotic cues. CPKs also protect growth by restoring cellular homeostasis following prolonged stress. For instance, CPK2/6/11 phosphorylate the ER-localized Ca^2+^ pump ECA1 to restore cytosolic Ca^2+^ homeostasis, preventing ABA overaccumulation and ensuring sustained root elongation (Liang et al., 2025). This mechanism highlights the role of CPKs in terminating stress signaling to avoid chronic inhibition of growth. However, the spatiotemporal regulation of these cascades and their underlying mechanisms require further investigation. We hypothesize that tissue-specific expression of these CPK isoforms enables roots and shoots to recover at different rates during long-term stress exposure, although this has not yet been experimentally verified. Collectively, these insights will help pinpoint CPKs that can be manipulated to improve plant stress resilience without causing growth penalties.

Despite extensive progress, many aspects of CPK function remain insufficiently understood. Given that CPKs participate in both stress signaling and developmental regulation, future studies should explore how isoform-specific activation thresholds or post-translational modifications fine-tune this balance. Understanding the role of CPKs in the growth-defense trade-off requires the development of innovative technologies capable of simultaneously monitoring calcium signaling, CPK activity, phytohormone levels, and growth parameters in real-time within living organisms and specific cell types. In particular, most functional studies have focused on abiotic stress adaptation, whereas research addressing biotic stresses remains limited. Current findings describe their spatiotemporal expression and kinase activity during immune responses, but fundamental questions remain unanswered. How Ca^2+^-CPK pathways interact with other kinases? how CPKs cooperate with additional signaling proteins to ensure signaling specificity, and how these networks ultimately determine resistance outcomes require further investigation? In addition, One outstanding question concerns the systemic propagation of immune signals. For instance, flg22 can move through vascular connections to distal tissues, but the mechanisms by which Ca^2+^ signals are transmitted to these distant cells and initiate defense remain unresolved (Jelenska et al., 2017). The electrical signaling, ROS waves, and hydraulic changes may act together with Ca^2+^ oscillations to mediate rapid long-distance communication. However, how CPKs integrate these distinct systemic signals remains a major open question that could reshape our understanding of systemic acquired resistance (Gilroy et al., 2016).

Future research on CPKs should prioritize a multi-scale, mechanistic dissection of their signaling roles. Progress requires moving beyond descriptive studies toward quantitative analyses that define the *in vivo* dynamics and functional outcomes of CPK activation. This will require developing of genetically encoded biosensors, such as CPK-specific FRET reporters, will be pivotal for real-time visualization of spatiotemporal decoding patterns (Xu et al., 2024). These tools, combined with precision genome editing approaches including CRISPR-mediated domain swapping and kinase-dead or phospho-mimetic mutagenesis, will enable functional mapping of the conserved motifs and autophosphorylation sites that determine signaling specificity and calcium sensitivity (Fu et al., 2025; Zou et al., 2025). Beyond these technological advances, incorporating parameters such as hetero-oligomerization, subcellular localization, and crosstalk with other decoder families into kinetic models will advance a more predictive understanding of plant information processing.

Ultimately, translating these mechanistic insights into practical applications will be crucial. Fine-tuning specific CPK alleles or regulatory modules represent a promising strategy for designing crops with durable, broad-spectrum resistance while maintaining optimal growth and yield. Thus, bridging fundamental discovery with agricultural innovation will require sustained efforts to unravel the sophisticated signaling calculus that enables CPKs to masterfully coordinate plant immunity and productivity.
